# Two Species Whose Ranges Are Shifting Uphill Are Not Pollen Limited, But Are More Successful Selfers Than Two Non‐Shifting Species

**DOI:** 10.1002/ece3.73429

**Published:** 2026-04-07

**Authors:** Inna Osmolovsky, Joshua M. Botti‐Anderson, Giancarlo M. Chiarenza, Zoe A. Xirocostas, Eve Slavich, Angela T. Moles

**Affiliations:** ^1^ Evolution & Ecology Research Centre School of Biological, Earth and Environmental Sciences, UNSW Sydney New South Wales Australia; ^2^ Fenner School of Environment and Society The Australian National University Canberra Australian Capital Territory Australia; ^3^ ARC Training Centre for Healing Country, Department of Molecular and Life Sciences Curtin University Perth Western Australia Australia; ^4^ School of Life Sciences University of Technology Sydney Sydney New South Wales Australia; ^5^ Stats Central, Mark Wainwright Analytical Centre, UNSW Sydney New South Wales Australia

**Keywords:** climate change, pollen deposition, pollen limitation, range shifts, reproductive success, selfing

## Abstract

Many species are expected to shift their ranges uphill in response to climate change and shifts to new ranges can be associated with changes in both positive and negative biotic interactions. We asked whether two uphill shifting species are more pollen limited at the upper range edge versus distribution core than are two non‐shifting species and whether the two uphill shifting species reproduce more successfully through self‐fertilisation (selfing) than the non‐shifting species. We performed pollinator exclusion, open pollination and pollen supplementation experiments and counted pollen grains deposited on the stigmas of six alpine plant species native to Kosciuszko National Park, NSW, Australia. Counter to our hypothesis, the uphill shifting species in our study did not experience pollen limitation in any part of their distribution, while non‐shifting species were more pollen limited in the core of their distribution than at range edges. However, the two uphill‐shifting species did show higher selfing rates than did non‐shifting species. Our results suggest that reproductive mechanisms may be non‐uniform across species ranges, although the spatial variability of traits is seldom taken into account in predictive models. Although plant‐pollinator interactions are often predicted to alter in the face of climate change, our study shows that some uphill‐shifting plants may be able to maintain sufficient pollinator services.

## Graphical abstract

Many plants are shifting their ranges uphill in response to climate change. During this process, they may lose their pollinators, facing decreased reproductive success. Surprisingly, we discovered that uphill shifting plants were not more pollen limited than non‐shifting plants and exhibited higher selfing success. Our results suggest that some range shifting plants may be able to maintain reproductive success in the face of climate change.

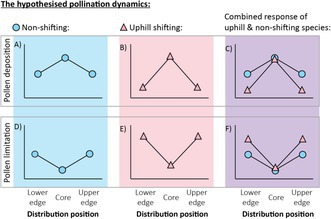

## 1 Introduction

With the onset of climate change, many species are shifting their ranges uphill and poleward (Lenoir et al. 2020; Osmolovsky et al. In review; Parmesan and Yohe 2003). In the process of shifting into a new range, species are expected to undergo changes in their biotic interactions—leaving some of their enemies and mutualists behind (Keane and Crawley 2002; Mitchell et al. 2006; Alpert 2006; Moles et al. 2022). The changes in biotic interactions expected with the onset of climate change have been investigated by surveying the extent of biotic interactions across species' ranges without consideration of possible range or phenological shifts (Hargreaves et al. 2015; Hillerislambers et al. 2013; Moeller et al. 2012; Rivest and Vellend 2018; Andrew et al. 2012) and by transplanting plants beyond their current distribution (Bektaş et al. 2024; Andrew and Hughes 2007; Brown and Vellend 2014). Both methods provide valuable insight into how biotic interactions may affect and be affected by range shifts. However, comparing the patterns of biotic interactions between shifting and non‐shifting species may allow us to isolate the impacts of range shifts on biotic interactions from the general edge effects and the effects of climate change (Fowler et al. 2023; Hargreaves et al. 2014). Further, exploring the extent of pollen limitation in non‐shifting species is crucial to understanding how pollination affects and is affected by range shifts in response to climate change. While non‐shifting species are not growing in isolation from climate change, they provide an important baseline for comparison with species that have shifted their ranges. In this study, we compared the extent of pollen limitation and selfing success patterns between uphill and non‐shifting species.

**FIGURE 1:**
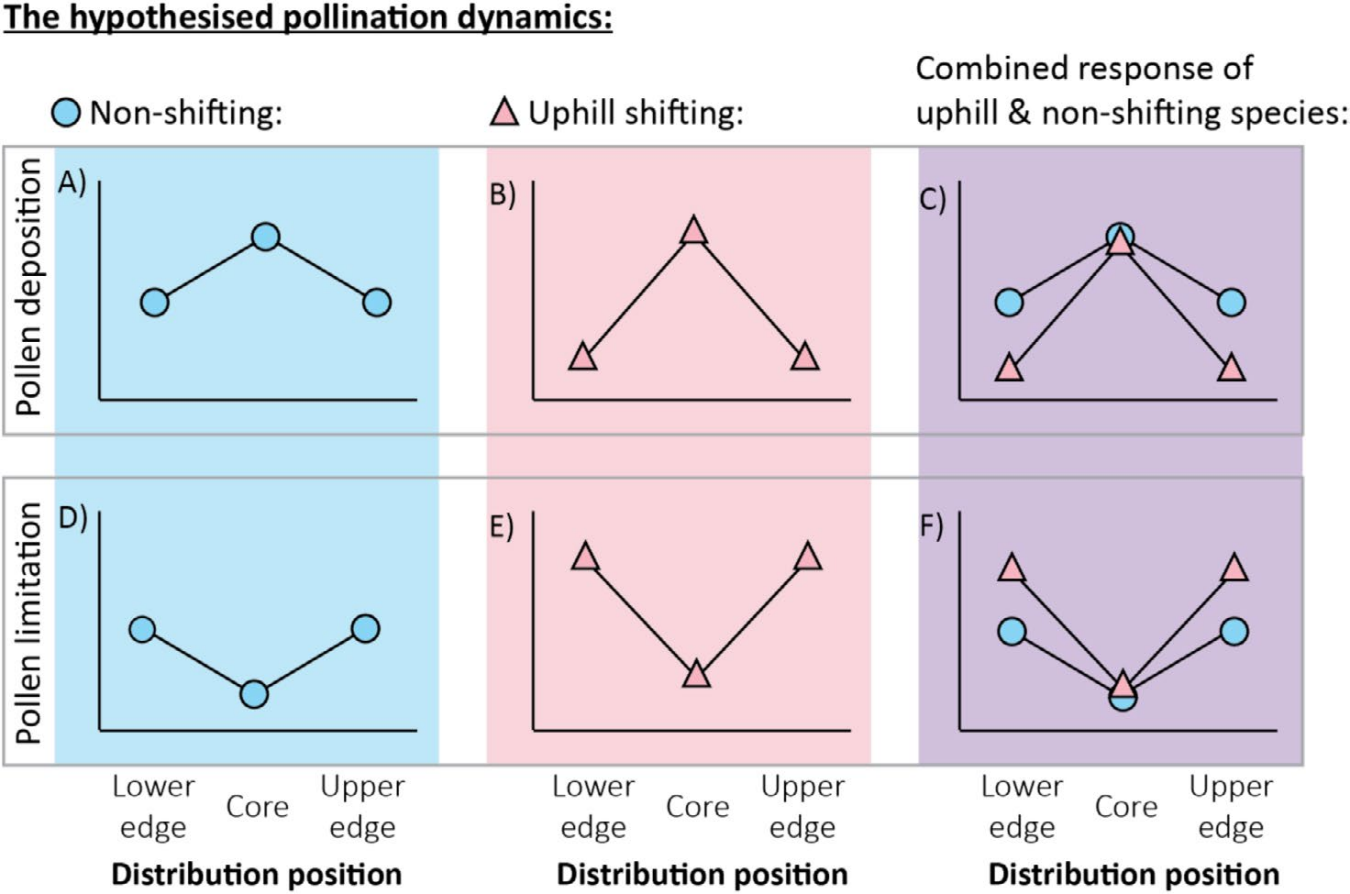
The graphic representation of the expected (A–C) pollen deposition and (D–F) pollen limitation at the lower range edge, distribution core and the upper range edge of shifting and non‐shifting species and the combined response of uphill and non‐shifting species.

Under the Abundant Centre Hypothesis, edge populations have fewer individuals compared to core populations (Brown et al. 1995; Sagarin and Gaines 2002), affecting the strength of the biotic interactions and the abundance of interacting organisms (Bullock et al. 2000; Louthan et al. 2015). At range edge, Allee effects (Stephens et al. 1999; Davis et al. 2004) may result due to fewer conspecifics leading to reduced pollinator visitation and pollen transfer (Evans et al. 2017; Roll et al. 1997). Studies exploring reproductive success across species ranges often focus on pollinator abundance and pollen limitation, but not pollen deposition (Koski et al. 2017, Benning and Moeller 2019, Theobald et al. 2016, Anderson et al. 2015, Baer and Maron 2018, Macrì et al. 2021, Anjum et al. 2025, Hillerislambers et al. 2013, Moeller et al. 2012, Rivest and Vellend 2018; but see: Hargreaves et al. 2015, Nathan and Gruner 2023, Chalcoff et al. 2012). Exploring the extent of pollen deposition can improve our understanding of the presence and efficiency of pollinators across species' ranges, providing a possible mechanism for any observed changes in reproductive success. We first hypothesise that species would experience lower pollen deposition at the upper (cold) and lower (warm) range edges than in their distribution cores (Figure 1A).

A low abundance of pollinators, that were associated with the plant species does not always result in pollen limitation. Specifically, some plants are able to reproduce through self‐fertilisation (Hargreaves et al. 2015; Mazzolari et al. 2017) or compensate for the loss of pollinators through life history, e.g., longer life spans or clonality (Carbognani et al. 2019; Munoz et al. 2016). Current evidence for changes in the extent of pollen limitation is mixed at different distribution positions, with eight studies suggesting no change in pollen limitation across species' ranges (Hargreaves et al. 2015; Koski et al. 2017; Benning and Moeller 2019; Theobald et al. 2016; Anderson et al. 2015; Baer and Maron 2018; Macrì et al. 2021; Nathan and Gruner 2023) and five supporting the hypothesised increase in pollen limitation toward range limits (Hillerislambers et al. 2013; Moeller et al. 2012; Rivest and Vellend 2018; Chalcoff et al. 2012; Anjum et al. 2025). One possibility is that this mixed evidence reflects the different strategies the plants in the various studies employ. For example, the plants were obligate outcrossers in four of the five studies reporting an increase in pollen limitation toward range edges (Chalcoff et al. 2012; Hillerislambers et al. 2013; Moeller et al. 2012; Rivest and Vellend 2018), while studies reporting no increase in pollen limitation had a mix of selfers and obligate outcrossers as study systems. One of the non‐shifting species in our study was reported as an obligate outcrosser (*Prasophyllum tadgellianum*; Freestone 2022), while the other species has been shown to have a strong association with pollinators (*Nematolepis ovatifolia*; Mitchell‐Storey 2024). Thus, we next test the hypothesis that southern hemisphere alpine species experience stronger pollen limitation at the upper and lower range edges than in their distribution cores (Figure 1D). None of the previous studies explored pollen limitation patterns across the ranges of plant species native to the Southern Hemisphere. However, pollination networks in the Australian Alps differ significantly from alpine ecosystems in the Northern Hemisphere (Inouye and Pyke 1988; Milla and Encinas‐Viso 2020). Consequently, understanding global patterns of pollination and reproductive success across species' ranges is crucial. The Missed Mutualist Hypothesis suggests that species that shift to a new range may suffer reduced positive interactions (Alpert 2006; Moles et al. 2022). The Missed Mutualist Hypothesis has mainly been discussed and supported in the context of introduced species invading novel ranges (Alpert 2006; Moles et al. 2022; Xirocostas et al. 2024). However, native species shifting their ranges in response to climate change may undergo a similar process, with plants potentially becoming pollen limited at their leading range edge by outpacing their preferred pollinators (Hegland et al. 2009; Morton and Rafferty 2017). Evidence on reproductive success in native range shifting plants comes from studies exploring pollen limitation across and beyond the distribution ranges of plants (Hargreaves et al. 2015; Hillerislambers et al. 2013; Moeller et al. 2012; Rivest and Vellend 2018) and reproductive success (but not pollen limitation) of range shifting species in situ and in silico (Dangremond and Feller 2016; Mazzolari et al. 2017; Memmott et al. 2007; Kennedy et al. 2021). However, none of these studies have explored pollen limitation patterns of species actively shifting their ranges in response to climate change or compared them to pollen limitation in non‐shifting species. We predict that the uphill shifting plants in our study would experience lower pollen deposition and be more pollen‐limited at their upper range edge vs. distribution core than the non‐shifting species (Figure 1C,F).

Uphill range shifts can result from the extinction of plants at the lower range edge, leading to lower edge contraction (Hampe and Petit 2005; Aitken et al. 2008; Brodie et al. 2025). Extinction may occur directly due to changes in abiotic conditions, such as local climate becoming unsuitable, which may directly affect species' fitness (Somero 2011). However, local extinction in response to climate change could also occur indirectly, for example, through climate change induced changes in biotic interactions, such as animal‐mediated pollination (Andrzejak et al. 2022; Cahill et al. 2013). For example, pollinating insects may disappear from the lower range edge, but not outpace uphill shifting plants at the upper range edge, for example, by shifting their phenology (e.g., Robbirt et al. 2014), or shifting their ranges downhill. The subsequently reduced pollination may cause low reproductive success and inbreeding depression, thereby increasing extinction risk and range contraction at the lower range edge (Carbognani et al. 2019; Moracho et al. 2016; Levin 2011), resulting in an overall uphill shift in distribution. Despite the importance of pollination and reproductive success, we are not aware of any previous studies that have quantified pollen deposition or pollen limitation at species' lower (or downhill) range edges of species that shifted their ranges in response to climate change. Thus, our next goal was to test the hypothesis that the uphill shifting species in our study experience lower pollen deposition and higher pollen limitation at the lower range edge vs. the distribution core than do non‐shifting species (Figure 1C,F).

**FIGURE 2:**
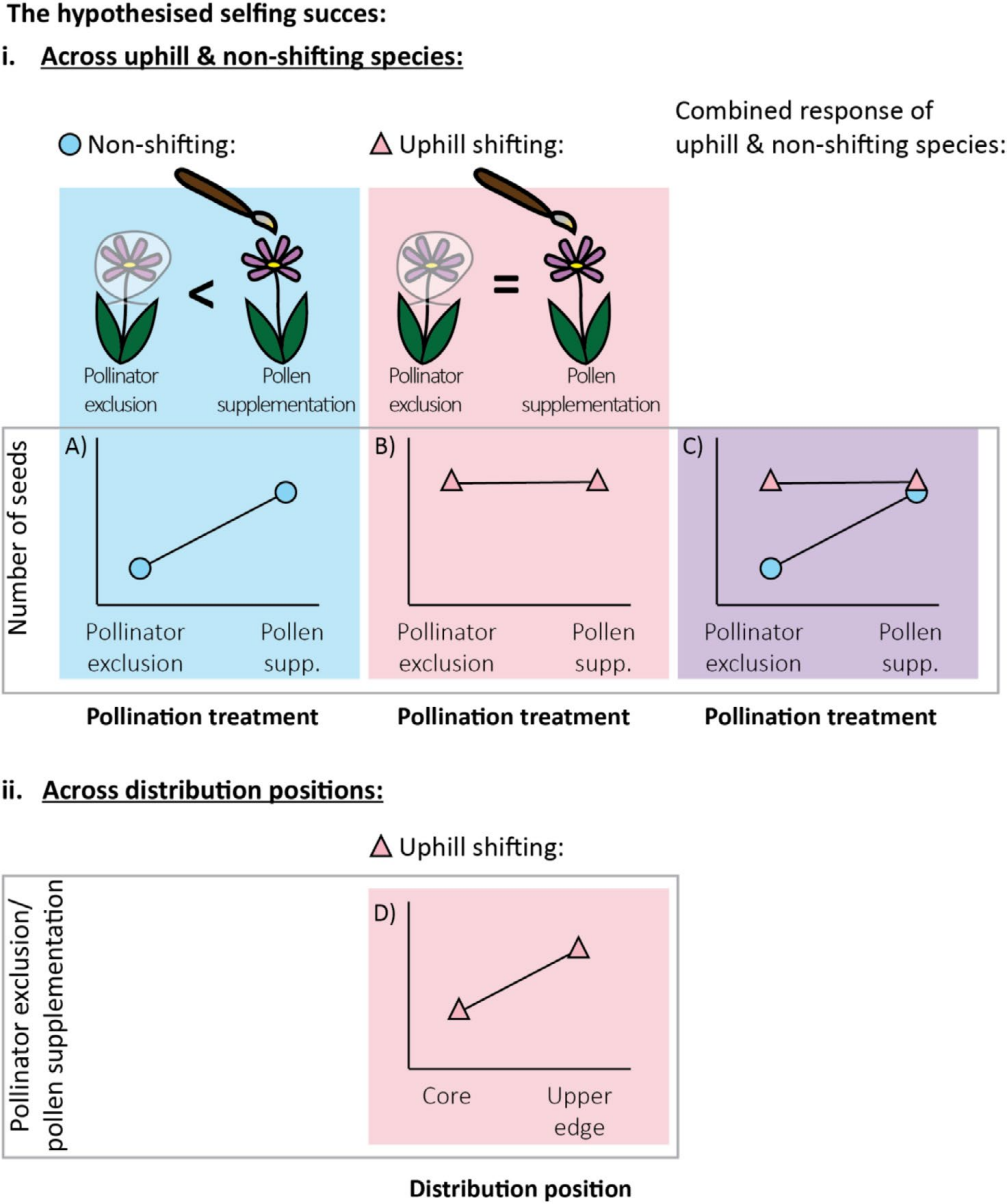
(i) The schematic comparison between selfing dynamics in shifting and non‐shifting species. Panels A–C display the hypothesised number of seeds resulting from pollinator exclusion and pollen supplementation treatments. (ii) The hypothesised selfing success dynamics across the distribution positions of uphill shifting species. Panel D displays the ratio of seeds resulting from pollinator exclusion versus pollen supplementation treatments at the distribution core and upper range edge.

Self‐fertilisation (selfing, hereafter) can compensate for the loss or absence of efficacious pollinators and the reduction in the abundance of mates, ensuring successful reproduction, dispersal and colonisation of novel niches (Pannell 2016; Eckert et al. 2010; Baker 1967; Kalisz and Vogler 2003; Fowler et al. 2023; Bond et al. 1994; Hargreaves and Eckert 2014). Thus, successful selfing has been hypothesised to be associated with greater niche breadth, invasion success of introduced species and with successful range shifts with the onset of climate change (Pannell 2016; Grant and Kalisz 2020). However, a data compilation study found no association between the rate of range shifts in native plants and their reproductive mechanisms (including outcrossing, mixed mating and selfing; Angert et al. 2011). Conversely, we were unable to identify studies that have tested the correlation between range shifts and selfing success. We hypothesise that the two uphill shifting species would be more successful selfers, producing a similar number of seeds from pollinator exclusion and pollen supplementation treatments than would the two non‐shifting species (Figure 2C).

Expanding populations are often faced with strong selective pressures in diverging directions. Plants at expanding range edges often develop high reproductive output that correlates with successful and long‐distance dispersal (Tomiolo and Ward 2018; Hampe 2011; Levin 2011). On the other hand, expanding populations are faced with the challenges of establishment in novel environments (Tomiolo and Ward 2018), the loss of pollinators (Memmott et al. 2007; Hegland et al. 2009) and Allee effects (Stephens et al. 1999; Davis et al. 2004). In combination, these factors are hypothesised to promote selection for higher seed production through selfing in the new ranges (Kalisz and Vogler 2003; Hargreaves and Eckert 2014; Baker 1955). Indeed, introduced plants have been empirically observed to develop selfing in their new ranges (Tabassum and Leishman 2019; Barrett et al. 2008; Rodger et al. 2013; Ward et al. 2012; Petanidou et al. 2012), with selfing especially prevalent at expanding range edges (Levin 2011). Further, expanding populations of native mangroves developed precocious reproduction, beginning reproduction at a younger age than core distribution populations (Dangremond and Feller 2016). These studies suggest that evolution can occur over relatively short time periods in response to both the introduction (Tabassum and Leishman 2019, Barrett et al. 2008, Rodger et al. 2013, Ward et al. 2012, Petanidou et al. 2012) and climate change (Dangremond and Feller 2016; Everingham et al. 2021). Thus, uphill shifting plants may reproduce through selfing at the range edges, but selfing might still be disadvantageous toward the distribution core, due to the abundance of potential mates and the need to reduce competition among individuals within populations (Jump and Peñuelas 2005). Thus, we predict that in the absence of pollinators, the two uphill shifting species would produce more seeds at their upper range edge than in their core (Figure 2D).

Native plants are already on the move. However, most of our hypotheses are based on theories and evidence from pollination dynamics and reproductive success experienced by non‐native species in their introduced ranges. Here, we extend this work by advancing understanding of the way native species are shifting their ranges in response to climate change also experience changes in reproductive success and pollination.

In summary, our hypotheses are:

*Plant species would experience lower pollen deposition at the upper (cold) and lower (warm) range edges than in their distribution cores*.

*The plant species in our study would experience stronger pollen limitation at the upper (cold) and lower (warm) range edges than in their distribution cores*.

*Uphill shifting plants would experience lower pollen deposition and be more pollen‐limited at their upper range edge* vs. *distribution core than do non‐shifting species*.

*Uphill shifting species would experience lower pollen deposition and higher pollen limitation at the lower range edge* vs. *the distribution core than do non‐shifting species*.

*Uphill shifting species would be more successful selfers than non‐shifting species*.

*Uphill shifting species would produce more seeds in the absence of pollinators at their upper range edge than in their core*.

## 2 Methods

### 2.1 Study Area

We conducted our study in alpine vegetation in Kosciuszko National Park, New South Wales, Australia (36.4317° S, 148.3289° E) between November 2023 and March 2024. Our study sites spanned from 1700 to 2210 m a.s.l. in elevation (see Appendix S1 for a full list of the sampled locations, their elevation and coordinates). Precipitation in the alpine zone (1850–2210 m a.s.l.; Costin et al. 1979) ranges from 1800 to 3100 mm per year, with 60% of the precipitation falling as snow (Green and Osborne 1994). Over the last 60 years, the area experienced an increase in annual temperatures and a decline in snow depth and the duration of snow cover (Hennessy et al. 2008; Bhend et al. 2012). The vegetation is characterised by low‐growing shrubs, grasses and forbs (Bear et al. 2006).

### 2.2 Species Selection

We chose six species from a study on the range shifts of 36 alpine plant species native to Kosciusko National Park (Auld et al. 2022). We chose three species that displayed significant uphill range shifts and three species that did not exhibit significant changes to their distribution. These species were easily distinguishable from other species, were flowering during the sampling season (late November–early March), with flowers that were easy to hand‐pollinate and were abundant at the three points of distribution we sampled. We preferentially selected species that were resurveyed during the 2020 survey.

Five of the six species in our study are monoecious with hermaphroditic flowers (Costin et al. 1979), with *Aciphylla glacialis* as the only dioecious species (Pickering 2000). While some information exists regarding the phenology and the visiting pollinators of some of the species (Mitchell‐Storey 2024; Milla and Encinas‐Viso 2020; Inouye and Pyke 1988), only two studies explored self‐compatibility in two of the species (*Prasophyllum tadgellianum* and *Pentachondra pumila*), suggesting that they might be obligate outcrossers (Freestone 2022; Godley 1966). However, these studies were conducted in the Alpine National Park in Victoria, Australia (Freestone 2022) and in New Zealand (Godley 1966) and regional differences in reproductive mechanisms might exist.

### 2.3 Pollination Experiments

We performed pollination treatments on four of the chosen species (Table 1), because *Aciphylla glacialis* and *Pappochroma setosum* mostly finished flowering by the beginning of the sampling period. We sampled three positions of each species' distribution: the upper and lower range edges and the distribution core (overall, we sampled 18 sites). Range edges were selected based on the lower‐most and upper‐most locations where each species was previously observed and recorded. We then checked if the species was present at the indicated locations. If we were unable to locate the species, we searched for lower/higher locations within an elevation range of 200 m of the lowermost and uppermost edges. We could not locate any individuals at a previously recorded lower edge for *Dichosciadium ranunculaceum* var. *ranunculaceum*, opting for a higher reported position as the new edge. Because our study focused on understanding reproductive dynamics of uphill and non‐shifting species, we excluded several species initially considered for the study, but for which we were unable to identify the upper edge population within 200 m of elevation from the indicated location. Core positions were chosen as close as possible to the centroid of the distribution. At each location, we ensured a minimum of 5 m distance between the sampled area and any trail or road and that at least 15 individuals were present, as some of the individuals were used as pollen donors and some individuals were used as pollen acceptors. All the edge positions we observed had at least 15 individuals.

**TABLE 1:** The list of the sampled species, their families, the direction of their shift according to Auld et al. (2022) and the reproduction indices measured for each species.

Species	Family	Shift direction	Measured reproduction indices
*Dichosciadium ranunculaceum* var. *ranunculaceum*	Apiaceae	Uphill 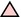	Pollen limitation Pollen deposition Selfing success
*Pappochroma setosum*	Asteraceae	Uphill 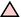	Pollen deposition
*Pentachondra pumila*	Ericaceae	Uphill 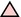	Pollen limitation Selfing success
*Aciphylla glacialis*	Apiaceae	No shift 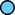	Pollen deposition
*Nematolepis ovatifolia*	Rutaceae	No shift 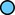	Pollen limitation Selfing success
*Prasophyllum tadgellianum*	Orchidaceae	No shift 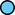	Pollen limitation Selfing success

At each site, we randomly chose 10 individuals as the target individuals for pollination treatments. We used a compass and a random number generator to assign direction and distance to the next individual, marking the closest plant in that direction and distance. We never backtracked our steps, ensuring a distance of at least a two‐canopy distance between one sampled individual and the next (2–10 m, depending on the species). We aimed to choose individuals with a mix of open and unopened buds, skipping individuals that were not flowering or only had senescent flowers. Next, we collected anthers or flowers bearing anthers from 5 to 20 donor individuals, each located at least 10 m away from the target individuals. The collected pollen was used for all subsequent pollen supplementation treatments.

On each of the target individuals, we performed 1–3 of the three possible pollination treatments (i.e., more than one pollination treatment was performed on each individual):

- Open pollination.
- Pollen supplementation. We applied pollen collected from nearby donor plants to the stigmas of open flowers, using a fine paintbrush (Arnell et al. 2021).
- Pollinator exclusion. We covered unopened buds with mesh bags (Figure 1d).

We covered the open pollination and pollen supplementation flowers or inflorescences (in *Prasophyllum tadgellianum*) with fine mesh bags, after the pollen was applied to the pollen supplementation treatment, to reduce the potential loss of fruits and seeds. In both the open pollination and pollen supplementation treatments, we ensured that flowers were fully open and were exposed to native pollinators prior to covering them with mesh bags. While we randomly chose and assigned open and pollen supplementation treatments to open flowers, in both cases, we visually ensured that the flowers were mature, with receptive stigmas. The number of treatments and replicates depended on the flower arrangement of each species. On shrubby plants (e.g., *Nematolepis ovatifolia*) and plants with flowers arranged in rosettes (e.g., *Dichosciadium ranunculaceum* var. *ranunculaceum*), we assigned treatments to three randomly chosen sections on the target plants. At each section, we covered the nearest unopened bud (or buds, if they were on the same branch). Next, we randomly assigned the open and pollen supplementation treatments to the nearest open flowers or flower‐bearing branches. If the plant was more than 1 m in diameter, we sampled within 1 m of the first point encountered when locating the 10 individuals. While there might be a difference in resource allocations between different flowers on the same individual (Diggle 1997), we randomly assigned flowers for each treatment. Further, using only one pollination treatment per individual would have introduced a between‐individual variability in seed number between the treatments.

In *Prasophyllum tadgellianum*, the flowers are arranged in a spike. We thus performed up to two treatments on each spike, marking the treatments by tying coloured ribbons below the flowers included in each treatment. Any unopened flowers on a stalk were assigned to a pollinator exclusion treatment. Because different flowers on the spike get different resource allocation, with flowers at the lower parts of the spike often getting more resources, which might affect the number of produced seeds (Diggle 1997), we randomly allocated the open pollination and pollen supplementation treatments to different parts of open, but not senescent flowers on each spike. However, in plants where the flowers at the top of the spike were unopened, we always assigned pollinator exclusion treatments to these plants. We are aware that this might skew the number of resulting seeds; however, we applied pollinator exclusion treatments to fully unopened spikes in all populations. Senescent and fruiting flowers were removed from the flowering stalks. We then covered the whole stalk with a mesh bag. We ensured that flowers could not touch each other by covering them with larger mesh bags that allowed good spatial separation between the flowers, thus avoiding any potential pollen transfer between different flowers on the same plant. For each target individual, we recorded the number of treatments and the overall number of flowers within each treatment.

We performed pollination treatments on 1950 flowers across 120 individuals of two uphill and two non‐shifting species. However, in some locations, we had fewer final replicates and treatments than the initial number of treatments performed because animals and park visitors removed or disturbed our samples, resulting in only 1254 fruits collected. Only 23 fruits remained at the distribution core of *Prasophyllum tadgellianum* and no replicates of the pollinator exclusion treatment remained at the lower edge of *Dichosciadium ranunculaceum* var. *ranunculaceum* (see https://doi.org/10.6084/m9.figshare.28561100 for a full list of assigned and collected treatments).

We collected the resulting fruits and seeds from all but one species approximately 1 month after pollination. We collected the fruits of *Pentachondra pumila* 2 months after pollination because fruits in this species take longer to reach maturity (Costin et al. 1979). Before the seeds in each collected fruit were counted, we stored them in paper bags, ensuring dry conditions. In *Prasophyllum tadgellianum*, we sampled the seeds in three randomly selected fruits from each treatment, after ensuring that the average number of seeds from the three fruits was close to the average number of seeds in each treatment.

In summary, the seeds from the collected treatments allowed us to test whether pollen limitation in all the sampled species at all the distribution positions, compare the overall selfing capacity between all the species (at all the positions) and compare the selfing capacity between the upper edge and core of uphill‐shifting species, but not the lower range edge.

### 2.4 Estimating the Number of Pollen Grains Deposited on Stigmas

We collected untreated stigmas from one species that was part of the pollen supplementation treatments (*Dichosciadium ranunculaceum* var. *ranunculaceum*) and two species (*Aciphylla glacialis* and *Pappochroma setosum*) that had set fruits by the time we were able to sample them (Table 1). We collected whole fruits, with stigmas still attached to them. We observed flowering in all three species from late November to early December and collected fruits in early January. Therefore, the time between pollen deposition and stigma collection was similar across species and distribution positions, suggesting that any post‐pollination pollen loss would have been comparable. We used the number of pollen grains deposited on the stigmas as a measure of pollen deposition (Panchen and Johnston 2018).

At each site, we randomly chose a direction and number of steps, sampling the closest individual to the final point. We repeated this process until ten individuals were sampled, never backtracking our steps and ensuring a distance of at least two canopies between individuals. We then collected three randomly chosen reproductive units (as fruits were arranged in inflorescences) from three random sections of the target plant. We continued the process until three, or all present, inflorescences were sampled. We did not detect fruits, flowers, or flower stalks at the lowest population (1870 m) of *Pappochroma setosum*. We thus did not record the reproductive output of this population and omitted it from the statistical analyses.

Collected stigmas were stored in 70% Ethanol at 5°C before pollen counts. We counted the pollen on up to four stigmas from each *Aciphylla glacialis* & *Dichosciadium ranunculaceum* var. *ranunculaceum* sampled individual and 15 stigmas in *P. setosum* individuals.

Pollen grains were stained according to the protocol described by Hiscock et al. (2002). We isolated the stigmas by cutting the excess flower and fruit tissue. We then placed the stigmas on a carrying slide and stained them using several drops of 0.6% (w/v) Aniline Blue (Glentham Life Sciences, UK). Stigmas were incubated with the stain for ~3 h before observation under an epifluorescence microscope (Olympus BX53F2, Japan), using a DAPI filter. We counted all visible pollen grains attached to the surface of the stigma (Figure 1d). In *Pappochroma setosum*, some pollen was also deposited on the petals of the individual flowers; we thus counted all visible pollen on the individual flower.

We collected observations of pollen grains on 625 stigmas across five locations of one non‐shifting and two uphill‐shifting species.

### 2.5 Statistical Analysis

All analyses were performed in R version 4.4.1 (R Core Team 2024), coupled with RStudio 2023.06 0.421 (Posit Team 2023).

We tested the extent of pollen limitation between distribution positions of shifting and non‐shifting species by comparing the number of seeds resulting from open pollination treatment to pollen supplementation. Additionally, we tested pollen deposition by comparing the number of pollen grains deposited on the stigmas of mature flowers that had set fruits at the different distribution positions. Finally, we compared pollinator exclusion and pollen supplementation treatments between shifting and non‐shifting species and between distribution positions of uphill shifting species. In all types of analyses, we built a Generalised Linear Mixed Effect Model, using the Template Model Builder (*glmmTMB*; Brooks et al. 2017).

To test if non‐shifting species experienced lower pollen deposition toward range edges than in the distribution core [H1], we used the number of pollen grains as the response variable, with position as the explanatory variable. We included a term for a random intercept for the sampled inflorescence nested within individual, but not the species, as it explained a low proportion of the variance (2 × 10^−9^) and the term caused model overfitting, likely due to the small number of species sampled. We fitted a negative binomial distribution to the overdispersed, count type dependent variable:

$$\begin{array}{c} \text{Number of pollen grains}\sim {\text{position}}_{\left[\text{upper edge or lower edge or core},\text{henceforth upper or lower or core}\right]}\\ \;+\left(1|\text{individual}/\text{inflorescence}\right),\text{family}=\text{Negative binomial} \end{array}$$

To test whether non‐shifting plants were more pollen limited at the upper and lower range edges, compared to the distribution core [H2], we used the number of seeds as the response variable and the interactions between treatment type and distribution position as the explanatory variable. We included random intercepts for the sampled individuals nested within species:

$$\begin{array}{c} \text{Number of seeds}\sim {\text{treatment}}_{\left[\text{open pollination or pollen supplementation},\text{henceforth open or}\;\mathrm{supp}\right]}\\ \;\times {\text{position}}_{\left[\text{upper edge or lower edge or core},\text{henceforth upper or lower or core}\right]}+\left(1|\text{species}/\text{individual}\right),\text{family}=\text{Negative binomial} \end{array}$$

For models comparing the number of seeds between open vs. supplementation treatments at the upper edge versus distribution core of uphill and non‐shifting species [H3], the number of seeds resulting from each treatment was the dependent variable. The interaction between the type of treatment, distribution position and whether the species were shifting was the explanatory variable. We included random intercept for the sampled individuals nested within species:

$$\begin{array}{c} \text{Number of seeds}\sim {\text{treatment}}_{\left[\text{open or}\;\mathrm{supp}\right]}\times {\text{position}}_{\left[\text{upper edge or core, henceforth upper or core}\right]}\\ \;\times {\text{shifting}}_{\left[\mathrm{yes}\;\text{or}\;\mathrm{no}\right]}+\left(1|\text{species}/\text{individual}\right),\text{family}=\text{Negative binomial} \end{array}$$

For models comparing pollen deposition at the upper edge vs. distribution core of uphill and non‐shifting species [H3], the number of pollen grains was the response variable. The interaction between distribution position and whether the species was shifting was the explanatory variable. We included a random intercept for the sampled inflorecence nested within individual, nested within species:

$$\begin{array}{c} \text{Number of pollen grains}\sim {\text{position}}_{\left[\text{upper or core}\right]}\times {\text{shifting}}_{\left[\text{uphill or non}-\text{shifting}\right]}\\ \;+\left(1|\text{species}/\text{individual}/\text{inflorescence}\right),\text{family}=\text{Negative binomial} \end{array}$$

We used similar models to compare the number of seeds and pollen grains between the lower range edge and the core of uphill and non‐shifting species [H4]. The only difference was the positions included in the model: core and lower range edge (rather than the upper range edge).

To test if shifting species were more successful selfers than non‐shifting species [H5], we assigned the number of seeds as the dependent variable, with pollination treatment (pollinator exclusion or pollen supplementation, calculated according to: Moodley et al. 2016, Lloyd and Schoen 1992) and shifting as the independent variables. We included a term for random effect for individuals nested within species:

$$\begin{array}{c} \text{Number of seeds}\sim {\text{treatment}}_{\left[\text{pollinator exclusion or pollen supplementation},\;\text{henceforth closed or}\;\mathrm{supp}\right]}\times {\text{shifting}}_{\left[\text{uphill or non}-\text{shifting}\right]}\\ \;+{\text{position}}_{\left[\text{lower or upper or core}\right]}+\left(1|\text{species}/\text{individual}\right),\text{family}=\text{Negative binomial} \end{array}$$

Finally, to test if shifting species were self‐fertilising more toward their upper range edge, compared to the core and lower range edge [H6], we used the number of seeds as our response variable and pollination treatment and population as the predictive variables. We included a random effect for the sampled individual nested within species:

$$\begin{array}{c} \text{Number of seeds}\sim {\text{treatment}}_{\left[\text{closed or}\;\mathrm{supp}\right]}\times {\text{population}}_{\left[\text{upper or core}\right]}\\ \;+\left(1|\text{individual}/\text{species}\right),\text{family}=\text{Negative binomial} \end{array}$$

For all models, we were interested in the interaction term between treatment, distribution position and/or if the species exhibited range shifts (see Appendix S2 for the complete results of the full models). We used type III ANOVA from the *car* package (Fox and Weisberg 2019) to report the *p* values of the categorical and interaction terms and calculate marginal and conditional *R* ^2^ values for all the models using the *easystats* package (Nakagawa and Schielzeth 2013; Lüdecke et al. 2022). The assumptions of linear distribution of residuals and homogeneity of the variability were visually inspected using the *Dharma* package (Hartig 2022). We used the *emmeans* package to perform post hoc comparisons between the positions and treatment types for each of the species (see Appendix S3 for full results; Lenth 2023). We additionally used the *emmeans* package to calculate the ratios of seed numbers across treatments, the number of pollen grains and the standard errors across distribution positions (Lenth 2023), using the following formulae:

$$\text{Pollen limitation}=1-\frac{\text{Number of seeds from open pollination}}{\text{Number of seeds from pollen supplementation}}$$

$$\text{Selfing success}=\frac{\text{Number of seeds from pollinator exclusion}}{\text{Number of seeds from pollen supplementation}}$$

For the pollen deposition index, we simply used the mean total number of pollen grains observed on each stigma.

We prepared our figures using the *ggplot2* package (Wickham 2016).

## 3 Results

**FIGURE 3:**
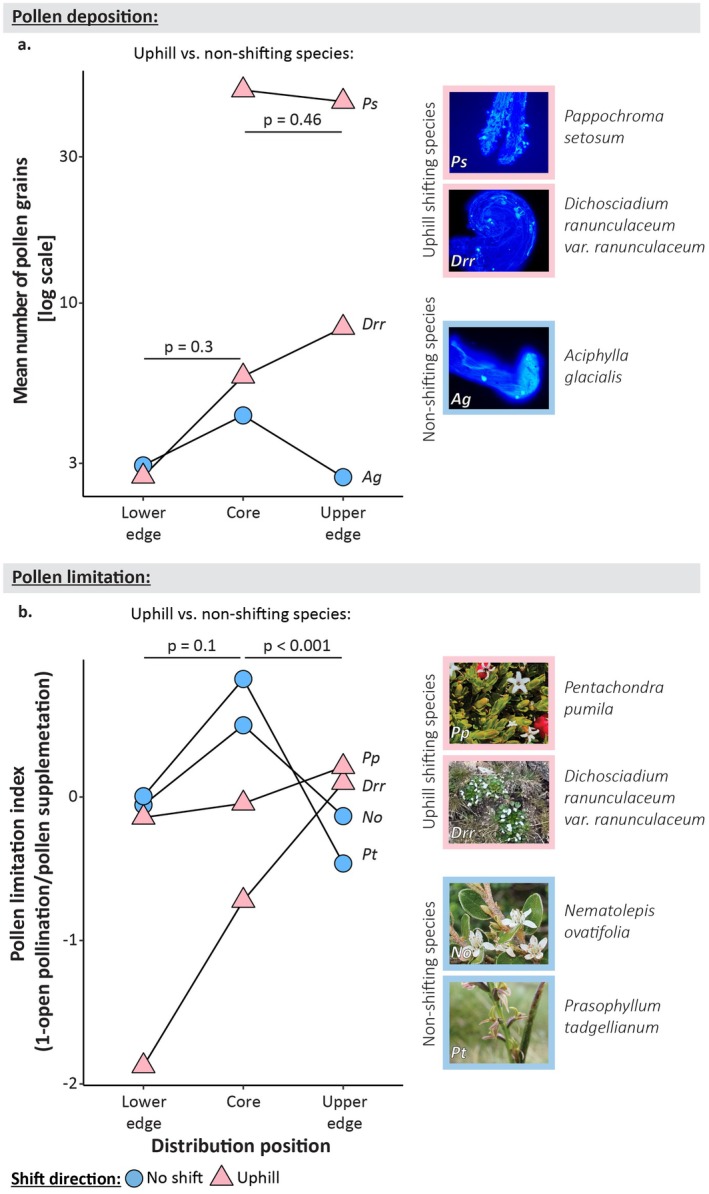
Pollen limitation across shifting and non‐shifting species. (A) The pollen deposition index and (B) the pollen limitation estimate, across three distribution positions of uphill (blue triangle) and non‐shifting species (pink circle). The triangles/circles denote the mean number of pollen grains for each species (A) and the seed ratio between open and pollen supplementation treatments of each species (B). The *p* values above the chart denote the significance of the interaction between distribution position and whether the species were shifting in (A) and the interaction between treatment, distribution position and whether the species were shifting in (B). The letters near the right triangles/circles denote the species the means are shown for, the first letter signifies the genus and the second the species and correspond with the species photos. The colour of the frame around each species photo signifies whether the species exhibited an uphill (pink) or no (blue) range shift. Photos Drr, Ps and Ag were taken by I. Osmolovsky, photo Pp was taken by A. T. Moles, photo No was taken by V. Williamson and photo Pt was taken by M. Mallen‐Cooper.

Contrary to our first hypothesis, the number of pollen grains did not substantially differ between the distribution positions of non‐shifting species (H1; *p* = 0.9, *R* ^2^ = 0.004; blue circles Figure 3a). That is, plants did not experience lower pollen deposition at the upper (cold) and lower (warm) range edges than in their distribution cores.

Contrary to our second hypothesis, the two non‐shifting species experienced higher levels of pollen limitation in their distribution cores than at distribution edges (H2; *p* = 0.01, *R* ^2^ = 0.01; Figure 3b), with plants in the distribution core producing substantially fewer seeds from open pollination than with pollen supplementation (seed ratio_[open vs supp]_ = 0.27, *p*_[open vs supp]_ = 0.006).

The two uphill shifting species did not experience higher levels of pollen limitation at the upper range edge relative to their distribution cores than did the non‐shifting species [H3]. There was a significant interaction between treatment_[open vs supp]_, distribution position_[upper vs core]_, and whether species were shifting (*p* < 0.001, *R* ^2^ = 0.11), but the effect was opposite to our initial prediction. The two non‐shifting species were pollen‐limited in the cores of their distributions (seed ratio_[open vs supp]_ = 0.28, *p*_[open vs supp]_ = 0.01). At all other positions, neither the uphill nor the non‐shifting species in our study experienced pollen limitation (seed ratio_[open vs supp]_ > 0.8; Figure 3b). However, plants at the distribution cores of the two uphill shifting species and the upper range edge of non‐shifting species unexpectedly produced fewer seeds from pollen supplementation than with open pollination (seed ratio_[open vs supp]_ > 1.3).

The two uphill shifting species did not experience lower pollen deposition at the upper range edges relative to the distribution cores than did non‐shifting species (H3; *p*_[uphill vs non‐shifting]_ = 0.46, *R* ^2^ = 0.3; Figure 3a).

Uphill shifting species did not experience higher levels of pollen limitation at their lower range edge relative to their distribution cores than did non‐shifting species (H4; *p* = 0.09, *R* ^2^ = 0.12; Figure 3b). The uphill shifting *Pentachondra pumila* and the non‐shifting *Nematolepis ovatifolia* did not exhibit any significant difference in pollen limitation across their ranges (seed ratio_[open vs supp]_ > 0.5, for both species; *P. pumila* : *p* = 0.82; *Nematolepis ovatifolia*: *p* = 0.44). On the other hand, the distribution core of the non‐shifting *Prasophyllum tadgellianum* was significantly more pollen‐limited (seed ratio_[open vs supp]_ = 0.2) than the lower range edge (seed ratio_[open vs supp]_ = 1.0, *p* = 0.02). The uphill shifting *Dichosciadium ranunculaceum var. ranunculaceum* produced more seeds from open pollination than pollen supplementation treatments at both the distribution core and lower range edge (seed ratio_[open vs supp]_ > 1.5); however, the difference was only statistically significant at the distribution core (*p*_[open vs supp]_ = 0.04), but not the lower range edge (*p* = 0.31).

The two uphill shifting species in our study did not experience lower pollen deposition at the lower range edges vs. their distribution cores than did the non‐shifting species (H4; *p*_[uphill vs non‐shifting]_ = 0.3, *R* ^2^ = 0.22; Figure 3a).

**FIGURE 4:**
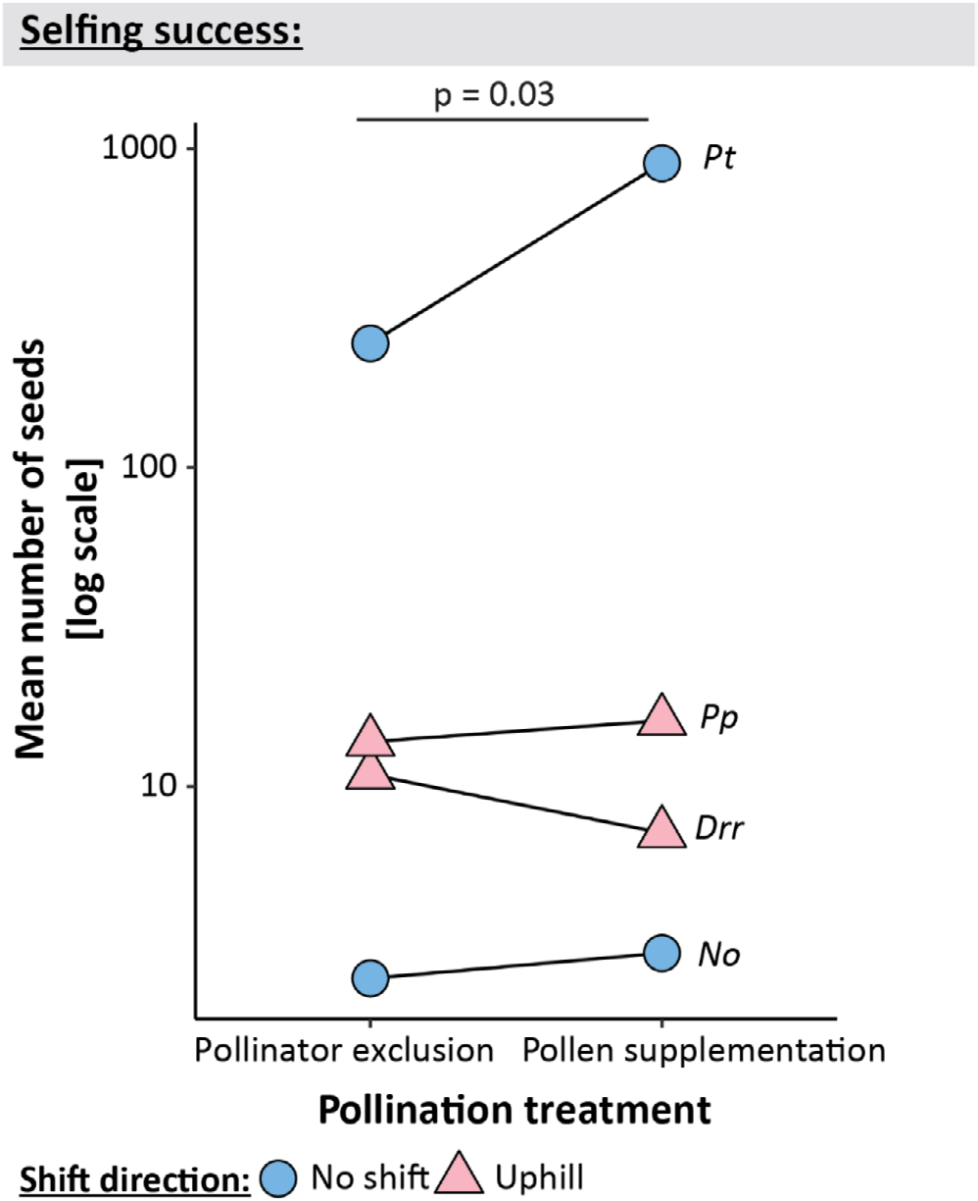
The potential of plants to produce seeds by selfing. The mean number of seeds produced from pollinator exclusion and pollen supplementation treatments for uphill (pink triangle) and non‐shifting (blue circle) species. Each triangle or circle denote the average number of seeds produced from pollinator exclusion or pollen supplementation treatments per species. The letters near the right triangles/circles denote the species the means are shown for, the first letter signifies the genus and the second the species.

None of the species in our study is an obligate outcrosser, as all four species at all distribution positions produced some seeds from the pollinator exclusion treatments (see Figure 4). Overall, there was no difference in the number of seeds produced from open pollination and pollen supplementation treatments (*p* = 0.45, *R* ^2^ = 0.02). In line with our fifth hypothesis, the two uphill shifting species were more successful selfers than were the two non‐shifting species (H5; *p* = 0.03, *R* ^2^ = 0.13; Figure 4). While the uphill shifting species in our study produced a seed ratio of 1 from pollinator exclusion compared to pollen supplementation (*p*_[closed vs open]_ = 0.97), the non‐shifting species had only a ratio of 0.4 (*p*_[closed vs open]_ = 0.02). This difference was primarily driven by the non‐shifting *Prasophyllum tadgellianum*, which produced a mean of 245 seeds from pollinator exclusion treatments, compared to 901 seeds from pollen supplementation. While the uphill shifting *Pentachondra pumila* also produced, on average, 1.1 times more seeds from open pollination than from pollinator exclusion treatment, the difference between the two treatments is much smaller than for *Prasophyllum tadgellianum*.

**FIGURE 5:**
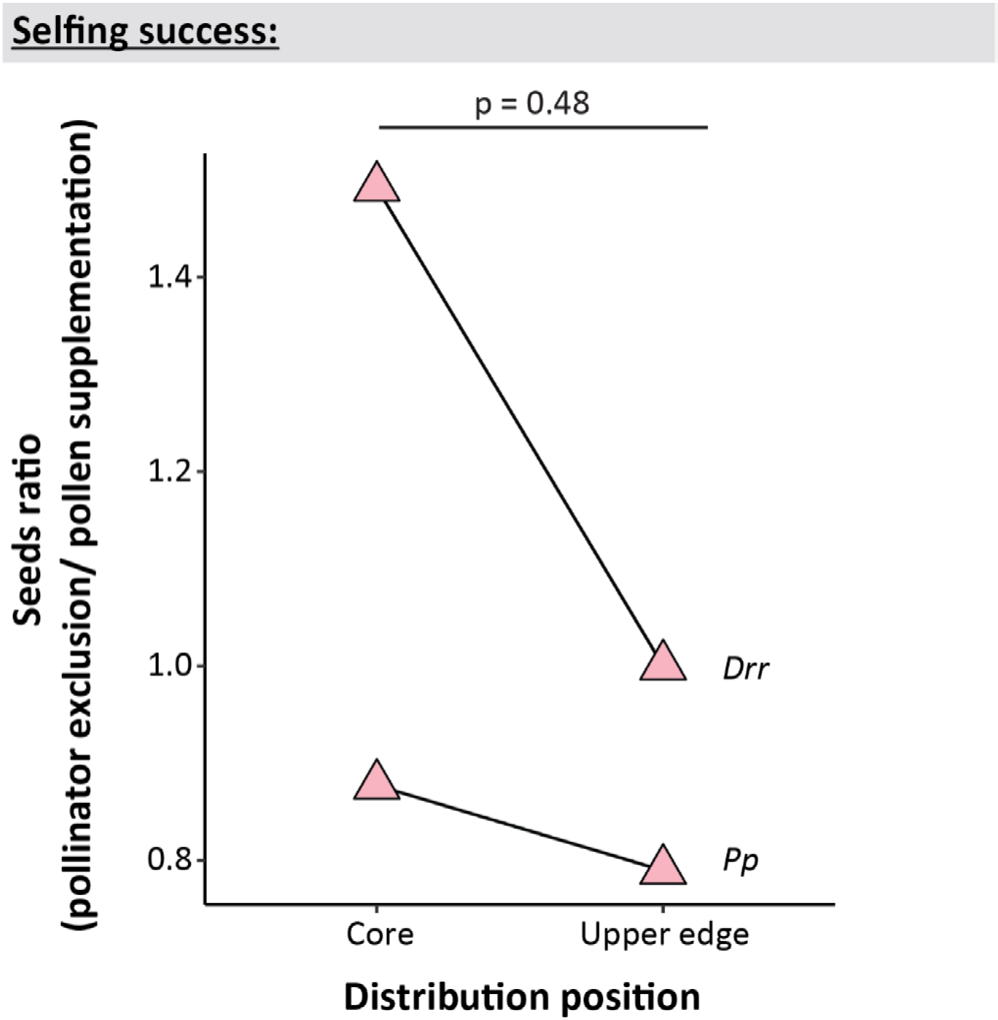
The ratio between seeds produced from pollinator exclusion and pollen supplementation treatments of uphill shifting species in the distribution core and upper range edge of each species. Each triangle denotes the ratio (pollinator exclusion/pollen supplementation) of seeds for each uphill shifting species. The *p* value above the chart denotes the significance of the interaction between pollination treatment and distribution position.

Contrary to our sixth hypothesis, neither of the two uphill shifting species in our study was a more successful selfer at the upper distribution edge than at their range cores (H6; *p* = 0.48, *R* ^2^ = 0.08; Figure 5). Both the uphill shifting *Dichosciadium ranunculaceum* var. *ranunculaceum* and *Pentachondra pumila* showed no significant difference in the closed to supplemented pollination treatments between the two distribution positions (*p* = 0.57 and 0.81, respectively).

In all our models comparing the number of seeds, > 90% of the variance was explained by the species random effect. These results reflect the fact that the different species in our study produce different numbers of seeds (< 10 for *Nematolepis ovitifolia*, > 60 for *Dichosciadium ranunculaceum var. ranunculaceum* and *Pentachondra pumila* and > 550 for *Prasophyllum tadgellianum*, per individual).

## 4 Discussion

Pollen limitation and low pollinator abundance have been predicted to be prominent at the leading edge of uphill shifting species (Hegland et al. 2009; Tomiolo and Ward 2018; Hargreaves and Eckert 2014), leading researchers to hold serious concerns about the ability of plants to continue to shift their ranges and survive in the face of climate change (Cahill et al. 2013; Hargreaves and Eckert 2014). While our study explored pollen limitation only in two uphill shifting species, neither of them suffered from reduced reproductive success or pollen limitation (Figure 3a,b). Our results might indicate a positive trend; however, reproduction may still fail beyond current range edges, as seeds also need to survive seed predation and establishment. Gaining an understanding of other biotic interactions will help to better understand species' overall vulnerability in the face of climate change. Further, exploring how range shifts impact reproductive success in other phylogenetically close shifting and non‐shifting plant species in multiple sites is important for a full understanding of the impacts of climate change. One possibility is that a stronger pattern of pollinator loss and reproductive failure at the leading edge of range shifting species would emerge, with sampling more shifting and non‐shifting species. It would also be interesting to test whether plants with different pollination vectors experience higher pollen limitation in response to climate change. Pollen limitation patterns may be dependent on the identity of the pollinating organisms, because most of the plants we sampled in our study are insect pollinated generalists (as is common in most alpine floras; Milla and Encinas‐Viso 2020, Inouye 2020), they may be less susceptible to alteration of pollinator communities than species pollinated by birds and mammals, as they are able to harness a large suite of pollinators (Bond et al. 1994).

The results in our study may be influenced by the small number of species sampled. We focused on sampling two uphill and two non‐shifting species at three distribution positions and conducted pollination experiments on 1950 flowers across 120 individuals. Thus, it might be possible that the patterns observed in our study do not reflect the general processes that shifting and non‐shifting species are undergoing with the onset of climate change, but stem from our species selection. However, we do have sufficient replication within each species to quantify the reproductive success across their ranges, testing if each species contradicts or conforms with our initial predictions. Our results show that a high proportion of the variability (> 90%) in our models is explained by species identity, suggesting that the identity of the species that were chosen in our study and their traits, may skew our conclusion. However, the high variability explained by species identity may stem from the fact that the different species produce different numbers of seeds (Bolker et al. 2009). We aimed to test the seed ratios between different treatments within the same species. Thus, our results reflect the dynamics of pollen limitation and selfing capacity between uphill and non‐shifting species. While the species in our study were phylogenetically unrelated, we selected species with similar reproductive traits. We chose plants with hermaphroditic flowers (Costin et al. 1979) with a selfing capacity (Figure 4), that are biotically pollinated (Freestone 2022; Inouye and Pyke 1988; Milla and Encinas‐Viso 2020; Mitchell‐Storey 2024), reducing the potential effect of the specific traits of the chosen species. However, the difference between shifting and non‐shifting species may stem from phylogenetic differences in traits. Conducting a phylogenetically informed study may provide a more robust understanding of range shifts on the reproductive success of plants. Nonetheless, most previous studies exploring the reproductive success of species shifting their ranges in response to climate change have sampled a single shifting species, with no comparison to the dynamics experienced by non‐shifting species (Dangremond and Feller 2016; Kennedy et al. 2021; Nathan and Gruner 2023). Further, these previous studies did not test pollen limitation and selfing success, which are important to the understanding of what might drive changes in reproductive success. Thus, despite including only four species, our study provides some important insights into the reproductive dynamics of species shifting their ranges in response to climate change.

Both the uphill‐shifting and non‐shifting plants in our study may be able to compensate, to a certain extent, for any loss of pollinators through self‐fertilisation, as both uphill and non‐shifting species produced seeds when pollinators were excluded (Figure 4). However, the two uphill‐shifting species in our study were more successful selfers than the two non‐shifting plants, producing almost an equal number of seeds from pollinator exclusion and pollen supplementation treatments (seed ratio = 0.9; Figure 4). This result is primarily driven by the lower selfing success in the non‐shifting *Prasophyllum tadgellianum*, suggesting that this result might be driven by the specific species sampled in this study. Nonetheless, our results are consistent with the idea that selfing might aid colonisation and establishment in a new range (Angert et al. 2011; Baker 1955; Barrett 2014; Grant and Kalisz 2020). Selfing may be advantageous if pollinator and mate abundance is low (Gibson‐Forty et al. 2022; Bond et al. 1994). Further, consistent with the selfing capacity observed in the non‐shifting species, selfing might offer an advantage for non‐shifting species, which, similarly to the uphill‐shifting plants, experience pollinator loss and environmental changes that may favour selfing (Bässler et al. 2013; Kalisz and Vogler 2003; Jones et al. 2013; Van Etten and Brunet 2013). On the other hand, selfing can be deleterious in the face of climate change, hindering adaptation to the changing environment (Stebbins 1957; Wright et al. 2013; Angert et al. 2020). We only tested the number of seeds produced. While the number of seeds may be a good indicator of successful fertilisation, selfing may prove disadvantageous at later life stages, hindering germination, growth, and reproduction (Charlesworth and Charlesworth 1987). However, compensating for the loss of pollinators may result in a higher fitness of range‐shifting plants than adaptation to novel conditions (Kubisch et al. 2013).

While the uphill shifting species in our study were overall more successful selfers than non‐shifting species, they exhibited lower selfing success at the upper range edge compared to the distribution core (Figure 5). This shows that species' reproductive mechanisms can be non‐uniform across their distribution. This finding suggests that plants in different parts of a species' distribution face differing selective pressures and thus may respond differently to climate change (Comte et al. 2014; Comte et al. 2024). Further, variation between distribution positions may be confounding some studies of species' responses to climate change. For instance, most studies testing for correlations between species traits (including reproductive mechanisms) and range shifts in response to climate change assume traits are uniform across species' ranges (Angert et al. 2011; Beissinger and Riddell 2021; Maclean and Beissinger 2017; Osmolovsky et al. In review). These studies often report weak relationships between the rate and direction of range shift and species' traits (Angert et al. 2011, Beissinger and Riddell 2021, Maclean and Beissinger 2017, Osmolovsky et al. In review). The findings from the two species in our study suggest that the within‐species variability in traits needs to be taken into account in order when predicting responses to climate change.

Contrary to our hypothesis, the two non‐shifting plants in our study experienced strong pollen limitation in the cores of their distribution, but not at distribution edges (Figure 3b). Interestingly, these results show a similar pattern to a previous study on alpine plants native to Kosciuszko National Park, where leaf damage was higher in the core of the species distribution than at the range edges (Osmolovsky et al. In press). Thus, neither herbivory nor pollination results are consistent with the hypothesised pattern in biotic interactions across species' distributions, or with the idea that biotic interactions limit species distributions (Louthan et al. 2015; Sexton et al. 2009). One possibility is that similar forces (such as pollinator and herbivore abundance and plant apparency and attractiveness: Haig and Westoby 1988, Ashman et al. 2004, Feeny 1976) govern both positive (pollination) and negative (herbivory) interactions in the same region. Another possibility is that while pollination and herbivory are different biotic interactions, they are taxonomically or functionally similar and thus may exhibit similar dynamics (e.g., a particular lepidopteran species might be an important herbivore and an important pollinator during different life phases). It would be worthwhile asking whether different biotic interaction types across different taxa show convergent patterns across species distributions in other locations.

In some locations, plants produced more seeds in the open pollination treatment than in the pollen supplementation treatment (Figure 3b). It is possible that we removed some of the previously deposited pollen during the pollen supplementation treatment, resulting in lower fertilisation and fewer seeds (Young and Young 1992). Alternatively, hand‐pollinations may have caused stigma damage, resulting in a smaller seed set (Young and Young 1992). However, if the pollen supplementation treatment had caused pollen removal or stigma damage, we would expect the same trend of higher open: pollen supplementation ratio across all distribution positions and this was not the case (Figure 3b). In some of the positions we sampled, many of the treatment replicates were removed by animals or visitors, resulting in a small sample size. However, the statistical tests account for sample size and we saw significantly higher seed sets in open treatments in multiple species and range positions, so it is highly unlikely to be a chance result. Another possibility is that this difference stems from a difference in resource allocation between flowers (Ashman et al. 2004), as we counted the number of seeds from each treatment per individual (rather than per a reproductive unit). Conversely, we selected the flowers for each treatment randomly and performed multiple treatments on each individual. A second possibility is that the difference in the success of open vs. pollen supplementation could stem from inbreeding depression. We collected pollen for the pollen supplementation treatment from individuals growing close (~10 m) to the target plants. If the genetic diversity in some locations is low, pollen originating from spatially close plants may lead to lower fertilisation rates and fewer seeds. The alteration of pollen movement and inbreeding depression have been hypothesised to increase with the onset of climate change, impacting both shifting and non‐shifting populations (Kremer et al. 2014; Kremer et al. 2012). However, more empirical studies are needed to understand the full effects of climate change on gene flow among different parts of species' distribution.

Climate change is already altering many aspects of our ecosystems—from phenology and spatial distribution to biotic interactions between organisms (Parmesan and Yohe 2003; Blois et al. 2013; Zeng et al. 2024). Despite changes in plant‐pollinator dynamics in the face of climate change, receiving relatively more attention than other biotic interactions (Hegland et al. 2009; Tomiolo and Ward 2018; Memmott et al. 2007; Hargreaves and Eckert 2014), empirical studies on the impact of range shifts on pollination and reproductive success are scarce. Our results suggest that, in some instances, range shifts may have surprisingly little effect on plant‐pollinator dynamics. We propose that other mutualistic interactions should also be investigated, as biotic interactions can have a critical impact on range shift success (Hargreaves and Eckert 2014; Osmolovsky et al. 2025).

## Author Contributions

**Inna Osmolovsky:** conceptualization (equal), formal analysis (lead), funding acquisition (supporting), investigation (lead), methodology (equal), project administration (lead), visualization (lead), writing – original draft (lead), writing – review and editing (equal). **Joshua M. Botti‐Anderson:** investigation (equal), methodology (equal), writing – review and editing (equal). **Giancarlo M. Chiarenza:** investigation (equal), methodology (equal), writing – review and editing (equal). **Zoe A. Xirocostas:** investigation (equal), methodology (equal), writing – review and editing (equal). **Eve Slavich:** formal analysis (equal), visualization (equal), writing – review and editing (equal). **Angela T. Moles:** conceptualization (equal), formal analysis (supporting), funding acquisition (lead), methodology (equal), supervision (lead), visualization (supporting), writing – review and editing (equal).

## Funding

This work was supported by Australian Research Council (DP180103611), Ecological Society of Australia, Student Research Award.

## Conflicts of Interest

The authors declare no conflicts of interest.

## Supporting information


**Appendix S1.** The sampled locations per species, their coordinates and altitude.
**Table S1a.** The list of all the locations of each species where we performed pollination experiments to assess pollen limitation and selfing success, the date when the pollination experiment was conducted and the fruit collection date.
**Appendix S2.** The full results of the statistical models.
**Table S2a.** The results and values of models comparing the number of seeds and pollen grains resulting from different pollination treatments across three distribution positions of uphill and non‐shifting species. The models are organised by the order of hypotheses (as described in the introduction). While to test our hypotheses we were interested only in the interaction terms, this table includes all tested terms.
**Appendix S3.** The results of the Generalised Linear Model analysis per species, and the results of the pairwise comparisons between pollination treatments and/or distribution positions.
**Table S3a.** Results of the Generalised Linear Model analyses of pollen limitation for each species (Hypotheses 2–4).
**Table S3b.** Pairwise comparison of pollen limitation between distribution positions for each species (Hypotheses 2–4).
**Table S3c.** Results of the Generalised Linear Model analyses of pollen deposition for each species (Hypotheses 1, 3–4).
**Table S3d.** Results of the Generalised Linear Model analyses of pollen deposition for each species (Hypothesis 5).
**Table S3e.** Results of the Generalised Linear Model analyses of selfing success at the upper range edge and distribution core for each uphill shifting species (Hypothesis 6).
**Table S3f.** Pairwise comparison of selfing success between distribution positions for uphill shifting species (Hypothesis 6).

## Data Availability

Code and data associated with this study are available at: https://doi.org/10.6084/m9.figshare.28561100.
