# Supplementary material for: Two Species Whose Ranges Are Shifting Uphill Are Not Pollen Limited, But Are More Successful Selfers Than Two Non‐Shifting Species
Source: Ecol Evol. 2026 Apr 7;16(4):e73429. doi: 10.1002/ece3.73429 (PMC13058242; doi:10.1002/ece3.73429)
Supplement: Supplementary file 1 — Appendix S1. The sampled locations per species, their coordinates and altitude. Table S1a. The list of all the locations of each species where we performed pollination experiments to assess pollen limitation and selfing success, the date when the pollination experiment was conducted and the fruit collection date. Appendix S2. The full results of the statistical models. Table S2a. The results and values of models comparing the number of seeds and pollen grains resulting from different pollination treatments across three distribution positions of uphill and non‐shifting species. The models are organised by the order of hypotheses (as described in the introduction). While to test our hypotheses we were interested only in the interaction terms, this table includes all tested terms. Appendix S3. The results of the Generalised Linear Model analysis per species, and the results of the pairwise comparisons between pollination treatments and/or distribution positions. Table S3a. Results of the Generalised Linear Model analyses of pollen limitation for each species (Hypotheses 2–4). Table S3b. Pairwise comparison of pollen limitation between distribution positions for each species (Hypotheses 2–4). Table S3c. Results of the Generalised Linear Model analyses of pollen deposition for each species (Hypotheses 1, 3–4). Table S3d. Results of the Generalised Linear Model analyses of pollen deposition for each species (Hypothesis 5). Table S3e. Results of the Generalised Linear Model analyses of selfing success at the upper range edge and distribution core for each uphill shifting species (Hypothesis 6). Table S3f. Pairwise comparison of selfing success between distribution positions for uphill shifting species (Hypothesis 6). [file ECE3-16-e73429-s001.docx]

**Supplementary material**

**Appendix S1.** The sampled locations of each species, their coordinates and elevation.

**Appendix S2.** The full results of the statistical models.

**Appendix S3.** The results of the Generalised Linear Model analysis per species, and the results of the pairwise comparisons between pollination treatments and/ or distribution positions.

**Appendix S1.** The sampled locations per species, their coordinates and altitude.

*Table S1a. The list of all the locations of each species where we performed pollination experiments to assess pollen limitation and selfing success, the date when the pollination experiment was conducted and the fruit collection date.*

| **Species** | **Position** | **Elevation (m)** | **Latitude** | **Longitude** | **Pollination date** | **Fruit collection date** |
| --- | --- | --- | --- | --- | --- | --- |
| *Dichosciadium ranunculaceum* var. *ranunculaceum* | Lower edge | 1870 | -36.40916667 | 148.3197222 | 6/01/2024 | 30/01/2024 |
| *Dichosciadium ranunculaceum* var. *ranunculaceum* | Core | 1940 | -36.41055556 | 148.3063889 | 6/01/2024 | 30/01/2024 |
| *Dichosciadium ranunculaceum* var. *ranunculaceum* | Upper edge | 2150 | -36.45611111 | 148.2669444 | 7/01/2024 | 31/01/2024 |
| *Nematolepis ovatifolia* | Lower edge | 1730 | -36.430833 | 148.322222 | 4/12/2023 | 9/01/2024 |
| *Nematolepis ovatifolia* | Core | 1840 | -36.431389 | 148.327778 | 4/12/2023 | 2/01/2024 |
| *Nematolepis ovatifolia* | Upper edge | 2060 | -36.450556 | 148.279444 | 6/12/2023 | 11/01/2024 |
| *Pentachondra pumila* | Lower edge | 1730 | -36.43027778 | 148.3194444 | 10/01/2024 | 1/03/2024 |
| *Pentachondra pumila* | Core | 2040 | -36.4475 | 148.3227778 | 5/01/2024 | 1/03/2024 |
| *Pentachondra pumila* | Upper edge | 2200 | -36.45722222 | 148.2633333 | 11/01/2024 | 2/03/2024 |
| *Prasophyllum tadgellianum* | Lower edge | 1720 | -36.43027778 | 148.3213889 | 3/01/2024 | 30/01/2024 |
| *Prasophyllum tadgellianum* | Core | 1950 | -36.44 | 148.3247222 | 2/01/2024 | 1/02/2024 |
| *Prasophyllum tadgellianum* | Upper edge | 2190 | -36.45444444 | 148.2627778 | 7/01/2024 | 31/01/2024 |

*Table S1b.* *The list of all the locations of each species where we collected stigmas for pollen availability.*

| **species** | **Position** | **Elevation** | **Latitude** | **Longitude** | **Stigma collection date** |
| --- | --- | --- | --- | --- | --- |
| *Aciphylla glacialis* | Lower edge | 1750 | -36.4283 | 148.31972 | 10/01/2024 |
| *Aciphylla glacialis* | Core | 2030 | -36.4781 | 148.27528 | 9/01/2024 |
| *Aciphylla glacialis* | Upper edge | 2200 | -36.4572 | 148.26306 | 11/01/2024 |
| *Dichosciadium ranunculaceum* var. *ranunculaceum* | Lower edge | 1870 | -36.4092 | 148.31972 | 6/01/2024 |
| *Dichosciadium ranunculaceum* var. *ranunculaceum* | Core | 1940 | -36.4106 | 148.30639 | 6/01/2024 |
| *Dichosciadium ranunculaceum* var. *ranunculaceum* | Upper edge | 2150 | -36.4561 | 148.26694 | 7/01/2024 |
| *Pappochroma setosum* | Lower edge | 1870 | -36.4117 | 148.32111 | NA |
| *Pappochroma setosum* | Core | 1960 | -36.4422 | 148.32528 | 4/01/2024 |
| *Pappochroma setosum* | Upper edge | 2090 | -36.4792 | 148.27333 | 9/01/2024 |

**Appendix S2.** The full results of the statistical models.

*Table S2a.* *The results and values of models comparing the number of seeds and pollen grains resulting from different pollination treatments across three distribution positions of uphill and non-shifting species. The models are organised by the order of hypotheses (as described in the introduction). While to test our hypotheses we were interested only in the interaction terms, this table includes all tested terms.*

| **Hypotheses** |  | **Value** | **SE** | **p-value** | **ANOVA p-value** | **R squared** |
| --- | --- | --- | --- | --- | --- | --- |
| **Hypotheses 1-2** | | | | | | |
| 1 - Pollen deposition at the core compared to edges of non-shifting species | Intercept | 0.94 | 0.26 | 0.00 | 0.00 | Conditional: |
|  | **Position - core** | **0.07** | **0.36** | **0.84** | **0.89** | 0.46 |
|  | Position - up | -0.10 | 0.36 | 0.78 |  | Marginal: |
|  | Inflorescence: individual | 0.01 |  |  |  | 0.00 |
|  | Individual | 0.52 |  |  |  |  |
| 2 - Pollen limitation at the core compared to edges of non-  shifting species | Intercept | 3.80 | 2.01 | 0.06 | 0.06 | Conditional: |
|  | Treatment - within | -0.02 | 0.26 | 0.93 | 0.93 | 0.98 |
|  | Position - core | -0.78 | 0.43 | 0.07 | 0.02 | Marginal: |
|  | Position – upper | 0.39 | 0.25 | 0.12 |  | 0.01 |
|  | **Treatment - within * position – core** | **1.32** | **0.54** | **0.01** | **0.01** |  |
|  | Treatment - within * position -up | -0.33 | 0.34 | 0.33 |  |  |
|  | Individual: species | 0.08 |  |  |  |  |
|  | Species | 8.03 |  |  |  |  |
| **Hypotheses 3-4** | | | | | | |
| 3 - Comparison between pollen limitation (pollen supp) at the cold edge of shifting and non-shifting species | Intercept | 3.04 | 1.51 | 0.04 | 0.04 | Conditional: |
|  | Treatment - within | 1.27 | 0.50 | 0.01 | 0.01 | 0.96 |
|  | Position – upper | 1.12 | 0.46 | 0.01 | 0.01 | Marginal: |
|  | Direction – up | -0.23 | 2.10 | 0.91 | 0.91 | 0.11 |
|  | Treatment - within * position – upper | -1.63 | 0.56 | 0.00 | 0.00 |  |
|  | Treatment - within * Direction - up | -1.50 | 0.54 | 0.01 | 0.01 |  |
|  | Position - upper * Direction – up | -1.86 | 0.54 | 0.00 | 0.00 |  |
|  | **Treatment - within * position - upper * direction - up** | **2.03** | **0.66** | **0.00** | **0.00** |  |
|  | Individual: species | 0.11 |  |  |  |  |
|  | Species | 4.23 |  |  |  |  |
| 4 - Comparison between pollen limitation (pollen supp) at the warm edge of shifting and non-shifting species | Intercept | 3.86 | 1.41 | 0.01 | 0.01 | Conditional: |
|  | Treatment - within | -0.02 | 0.33 | 0.96 | 0.96 | 0.93 |
|  | Position - core | -0.82 | 0.51 | 0.10 | 0.10 | Marginal: |
|  | Direction - up | -1.82 | 2.00 | 0.36 | 0.36 | 0.12 |
|  | Treatment - within * position - core | 1.32 | 0.66 | 0.05 | 0.05 |  |
|  | Treatment - within * Direction - up | -0.21 | 0.49 | 0.67 | 0.67 |  |
|  | Population - core * Direction - up | 1.66 | 0.59 | 0.00 | 0.00 |  |
|  | **Treatment - within * position - core * direction - up** | **-1.35** | **0.79** | **0.09** | **0.09** |  |
|  | Individual: species | 0.06 |  |  |  |  |
|  | Species | 3.89 |  |  |  |  |
| 3 - Comparison between pollen deposition at the cold edge of shifting and non-shifting species | Intercept | 1.01 | 0.89 | 0.26 | 0.26 | Conditional: |
|  | Position – upper | -0.18 | 0.35 | 0.60 | 0.60 | 0.79 |
|  | Direction - up | 1.69 | 1.10 | 0.12 | 0.12 | Marginal: |
|  | **Position - upper * direction - up** | **0.32** | **0.44** | **0.46** | **0.46** | 0.30 |
|  | Inflorescence: individual: species | 0.09 |  |  |  |  |
|  | Individual: species | 0.43 |  |  |  |  |
|  | Species | 0.73 |  |  |  |  |
| 4 - Comparison between pollen deposition at the warm edge of shifting and non-shifting species | Intercept | 0.86 | 1.00 | 0.39 | 0.39 | Conditional: |
|  | Position - core | 0.12 | 0.37 | 0.74 | 0.74 | 0.82 |
|  | Direction - up | 1.01 | 1.26 | 0.42 | 0.42 | Marginal: |
|  | **Position - core * direction - up** | **0.62** | **0.60** | **0.30** | **0.30** | 0.22 |
|  | Inflorescence: individual: species | 0.23 |  |  |  |  |
|  | Individual: species | 0.47 |  |  |  |  |
|  | Species | 0.92 |  |  |  |  |
| **Hypotheses 5-6** | | | | | | |
| 5 - Comparison of overall selfing in shifting and non-shifting species | Intercept | 2.65 | 1.47 | 0.07 | 0.07 | Conditional: |
|  | Position - core | 0.75 | 0.23 | 0.00 | 0.00 | 0.95 |
|  | Position - upper | 0.32 | 0.22 | 0.14 |  | Marginal: |
|  | Treatment - within | 1.01 | 0.43 | 0.02 | 0.02 | 0.13 |
|  | Direction - up | -0.81 | 2.03 | 0.69 | 0.69 |  |
|  | **Treatment - within * direction - up** | **-1.00** | **0.46** | **0.03** | **0.03** |  |
|  | Individual: species | 0.10 |  |  |  |  |
|  | Species | 3.94 |  |  |  |  |
| 6 - Selfing at the cold range edge of shifting plants | Intercept | 2.66 | 0.35 | 0.00 | 0.00 | Conditional: |
|  | Treatment - within | -0.07 | 0.24 | 0.77 | 0.77 | 0.54 |
|  | Position – upper | -0.56 | 0.32 | 0.08 | 0.08 | Marginal: |
|  | **Treatment - within * position - core** | **0.25** | **0.36** | **0.48** | **0.48** | 0.08 |
|  | Individual: species | 0.11 |  |  |  |  |
|  | Species | 0.15 |  |  |  |  |

**Appendix S3.** The results of the Generalised Linear Model analysis per species, and the results of the pairwise comparisons between pollination treatments and/ or distribution positions.

*Table S3a. Results of the Generalised Linear Model analyses of pollen limitation for each species (Hypotheses 2-4).*

| **Species** | **Term** | **Chisq** | **Df** | **Pr(>Chisq)** |
| --- | --- | --- | --- | --- |
| *Dichosciadium ranunculaceum* var. *ranunculaceum* | (Intercept) | 18.75 | 1.00 | 0.00 |
| *Dichosciadium ranunculaceum* var. *ranunculaceum* | Position | 8.22 | 2.00 | 0.02 |
| *Dichosciadium ranunculaceum* var. *ranunculaceum* | Treatment type | 1.03 | 1.00 | 0.31 |
| *Dichosciadium ranunculaceum* var. *ranunculaceum* | Position: treatment type | 0.95 | 2.00 | 0.62 |
| *Nematolepis ovatifolia* | (Intercept) | 13.16 | 1.00 | 0.00 |
| *Nematolepis ovatifolia* | Position | 2.24 | 2.00 | 0.33 |
| *Nematolepis ovatifolia* | Treatment type | 0.13 | 1.00 | 0.72 |
| *Nematolepis ovatifolia* | Position: treatment type | 1.63 | 2.00 | 0.44 |
| *Pentachondra pumila* | (Intercept) | 86.98 | 1.00 | 0.00 |
| *Pentachondra pumila* | Position | 3.46 | 2.00 | 0.18 |
| *Pentachondra pumila* | Treatment type | 0.10 | 1.00 | 0.75 |
| *Pentachondra pumila* | Position: treatment type | 0.40 | 2.00 | 0.82 |
| *Prasophyllum tadgellianum* | (Intercept) | 914.09 | 1.00 | 0.00 |
| *Prasophyllum tadgellianum* | Position | 7.21 | 2.00 | 0.03 |
| *Prasophyllum tadgellianum* | Treatment type | 0.00 | 1.00 | 0.98 |
| *Prasophyllum tadgellianum* | Position: treatment type | 8.35 | 2.00 | 0.02 |

*Table S3b. Pairwise comparison of pollen limitation between distribution positions for each species (Hypotheses 2-4).*

| **Species** | **Contrast** | **Position** | **Ratio** | **SE** | **df** | **null** | **z.ratio** | **p.value** |
| --- | --- | --- | --- | --- | --- | --- | --- | --- |
| *Dichosciadium ranunculaceum* var. *ranunculaceum* | open / supp | Lower | 2.09 | 1.51 | Inf | 1 | 1.01 | 0.31 |
| *Dichosciadium ranunculaceum* var. *ranunculaceum* | open / supp | Core | 1.53 | 0.31 | Inf | 1 | 2.06 | 0.04 |
| *Dichosciadium ranunculaceum* var. *ranunculaceum* | open / supp | Upper | 1.03 | 0.44 | Inf | 1 | 0.06 | 0.95 |
| *Nematolepis ovatifolia* | open / supp | Lower | 1.17 | 0.50 | Inf | 1 | 0.36 | 0.72 |
| *Nematolepis ovatifolia* | open / supp | Core | 0.46 | 0.29 | Inf | 1 | -1.21 | 0.23 |
| *Nematolepis ovatifolia* | open / supp | Upper | 1.09 | 0.39 | Inf | 1 | 0.24 | 0.81 |
| *Pentachondra pumila* | open / supp | Lower | 1.12 | 0.41 | Inf | 1 | 0.31 | 0.75 |
| *Pentachondra pumila* | open / supp | Core | 1.01 | 0.37 | Inf | 1 | 0.03 | 0.98 |
| *Pentachondra pumila* | open / supp | Upper | 0.79 | 0.33 | Inf | 1 | -0.56 | 0.58 |
| *Prasophyllum tadgellianum* | open / supp | Lower | 0.99 | 0.31 | Inf | 1 | -0.02 | 0.98 |
| *Prasophyllum tadgellianum* | open / supp | Core | 0.18 | 0.12 | Inf | 1 | -2.50 | 0.01 |
| *Prasophyllum tadgellianum* | open / supp | Upper | 1.49 | 0.43 | Inf | 1 | 1.39 | 0.17 |

*Table S3c. Results of the Generalised Linear Model analyses of pollen deposition for each species (Hypotheses 1, 3-4).*

| **Species** | **term** | **Chisq** | **Df** | **Pr(>Chisq)** |
| --- | --- | --- | --- | --- |
| *Pappochroma setosum* | (Intercept) | 884.45 | 1 | 0.00 |
| *Pappochroma setosum* | Position | 0.20 | 1 | 0.66 |
| *Dichosciadium ranunculaceum* var. *ranunculaceum* | (Intercept) | 11.16 | 1 | 0.00 |
| *Dichosciadium ranunculaceum* var. *ranunculaceum* | Position | 7.56 | 2 | 0.02 |
| *Aciphylla glacialis* | (Intercept) | 13.65 | 1 | 0.00 |
| *Aciphylla glacialis* | Position | 0.23 | 2 | 0.89 |

*Table S3d. Results of the Generalised Linear Model analyses of pollen deposition for each species (Hypothesis 5).*

| **Species** | **Term** | **Chisq** | **Df** | **Pr(>Chisq)** |
| --- | --- | --- | --- | --- |
| *Dichosciadium ranunculaceum* var. *ranunculaceum* | (Intercept) | 64.29 | 1 | 0.00 |
| *Dichosciadium ranunculaceum* var. *ranunculaceum* | Treatment type | 0.85 | 1 | 0.36 |
| *Nematolepis ovatifolia* | (Intercept) | 2.54 | 1 | 0.11 |
| *Nematolepis ovatifolia* | Treatment type | 0.20 | 1 | 0.65 |
| *Pentachondra pumila* | (Intercept) | 238.97 | 1 | 0.00 |
| *Pentachondra pumila* | Treatment type | 0.50 | 1 | 0.48 |
| *Prasophyllum tadgellianum* | (Intercept) | 540.36 | 1 | 0.00 |
| *Prasophyllum tadgellianum* | Treatment type | 16.22 | 1 | 0.00 |

*Table S3e. Results of the Generalised Linear Model analyses of selfing success at the upper range edge and distribution core for each uphill shifting species (Hypothesis 6).*

| **Species** | **Term** | **Chisq** | **Df** | **Pr(>Chisq)** |
| --- | --- | --- | --- | --- |
| *Dichosciadium ranunculaceum* var. *ranunculaceum* | (Intercept) | 81.67 | 1 | 0.00 |
| *Dichosciadium ranunculaceum* var. *ranunculaceum* | Treatment type | 1.01 | 1 | 0.32 |
| *Dichosciadium ranunculaceum* var. *ranunculaceum* | Position | 2.49 | 1 | 0.11 |
| *Dichosciadium ranunculaceum* var. *ranunculaceum* | Treatment type: position | 0.32 | 1 | 0.57 |
| *Pentachondra pumila* | (Intercept) | 150.04 | 1 | 0.00 |
| *Pentachondra pumila* | Treatment type | 0.20 | 1 | 0.66 |
| *Pentachondra pumila* | Position | 1.53 | 1 | 0.22 |
| *Pentachondra pumila* | Treatment type: position | 0.06 | 1 | 0.81 |

*Table S3f. Pairwise comparison of selfing success between distribution positions for uphill shifting species (Hypothesis 6).*

| **Species** | **Contrast** | **Position** | **ratio** | **SE** | **df** | **null** | **z.ratio** | **p.value** |
| --- | --- | --- | --- | --- | --- | --- | --- | --- |
| *Dichosciadium ranunculaceum* var. *ranunculaceum* | closed / supp | Core | 1.25 | 0.27 | Inf | 1 | 1.01 | 0.32 |
| *Dichosciadium ranunculaceum* var. *ranunculaceum* | closed / supp | Upper | 0.93 | 0.44 | Inf | 1 | -0.15 | 0.88 |
| *Pentachondra pumila* | closed / supp | Core | 0.88 | 0.26 | Inf | 1 | -0.44 | 0.66 |
| *Pentachondra pumila* | closed / supp | Upper | 0.79 | 0.24 | Inf | 1 | 0.78 | 0.43 |
